# Impression Cytology in Different Types of Contact Lens Users

**Published:** 2015

**Authors:** Guzin ISKELELI, Ceyhun ARICI, Mustafa DEGER BILGEC, Cuyan DEMIRKESEN, Hilal SERAP ARSLAN

**Affiliations:** 1 Istanbul University, Cerrahpasa Medical Faculty, Department of Ophthalmology, Turkey; 2 Eskisehir Medical Faculty, Department of Ophthalmology, Turkey; 3 Istanbul University, Cerrahpasa Medical Faculty, Department of Pathology, Turkey; 4 Kanuni Sultan Suleyman Training and Research Hospital, Department of Pathology, Turkey

**Keywords:** Contact Lens, Goblet Cells, Impression Cytology

## Abstract

This study compared tear function tests and cytologic changes on the conjunctival surface in asymptomatic patients wearing contact lens of different materials. Included in this study were 40 eyes wearing daily wear 4 week replacement hydrogel (H) lenses, 32 eyes wearing silicone hydrogel (SiH) lenses, 18 eyes wearing rigid gas-permeable (RGP) lenses, and 21 healthy eyes (no lenses) as the control group. Epithelial morphology of the conjunctival surface was evaluated, based on Nelson classification with conjunctival impression cytology (CIC), after the tear break-up time (TBUT) and Schirmer test were performed. The mean values of the Schirmer and TBUT tests were significantly higher in the control group than in the other lens groups (p < 0.001). Grade 0 was the most frequent CIC in the control group (66.7%) and least frequent in the SiH lens group (40.6%); grade I was least frequent in the control and RGP groups (33.3%) and most frequent in the SiH lens group (40.6%). Moreover, grade 2 was most frequent in the SiH lens group (18.8%). There was no statistically significant difference in goblet cell densities between the groups (p = 0.462). In addition to the different Schirmer and TBUT test results between contact lens wearers and healthy non-wearers, some cytologic changes may occur on the ocular surface with direct mechanical effects of contact lenses. This simple and noninvasive technique may be used to evaluate the ocular surface with regard to intolerance to contact lenses.

## INTRODUCTION

The tear film and the ocular surface are two important factors that influence a patient’s ability to wear contact lenses (1). Conjunctival impression cytology (CIC) is a simple noninvasive method for examining the conjunctiva’s suitability for contact lenses (2).

Conjunctival epithelial changes have been reported in symptomatic and asymptomatic lens wearers (3). The main changes are squamous metaplasia, decreased goblet cell density, and abnormal chromatin material in epithelial cell nuclei (4-6). These changes occur in dry eye syndromes.

The purpose of this study was to compare tear function tests and cytologic changes on the conjunctival surface in asymptomatic individuals using daily wear 4-week replacement hydrogel (H), silicone hydrogel (SiH), or rigid gas-permeable (RGP) contact lenses with the conjunctival surface of individuals with healthy eyes who did not use contact lenses.

## MATERIALS AND METHODS

Forty eyes of 20 individuals using 4-week replacement H lenses (Contact Day 30, Zeiss, Germany), 32 eyes of 16 individuals wearing SiH lenses (Air-Optix,Ciba-Vision, Duluth, GA, USA), 18 eyes of nine individuals wearing RGP lenses (A 90, Zeiss, Germany), and 21 healthy eyes of 21 individuals were included in the study. Epithelial morphology of the conjunctival surface was evaluated, based on Nelson grading (7) with CIC; after tear break-up time (TBUT), the Schirmer test was performed. All patients signed an informed consent document. This study was conducted in accordance with the tenets of the Helsinki Declaration. Sixty minutes after a patient totally removed the contact lenses, the TBUT and Schirmer tests were performed. Fluorescein smear was performed according to the TBUT after three or four cycles of eye opening and closing. Slit lamp biomicroscopy with a cobalt blue filter was employed to investigate the tear film layer. The interval from the last blink to appearance of the first dry spot on the cornea was determined with three repeats. The mean value was then calculated. For the Schirmer test, filter paper was used at the lateral one-third of the lower eyelid. Dampness from the eyelid was measured after 5 minutes. A drop of topical anesthetic was applied to the conjunctiva before the CIC procedure. After that, a 5- x 5-mm cellulose acetate filter paper with a 0.22-µm pore size was pressed with the dull side down for 3-4 seconds to the superior bulbar conjunctival surface at the 12 o’clock position, approximately 2 mm away from the limbus. The filter paper was then fixed in a solution of 75% acetone, 18.75% ethanol, 6.25% methanol in the recommended standard volume ratio 12:3:1 for 20 minutes. It was subsequently stained with periodic acid–Schiff. The sample was viewed under a light microscope at a magnification of 400x. Mean goblet cell densities were recorded per square millimeter. The goblet cell density, nucleus-to-cytoplasm ratio, and epithelial cell morphology were compared and graded by the Nelson grading system (7).

Statistical Analysis

The data were statistically analyzed with IBM SPSS Statistics Standard Pack 21, (Licensing Type: Network, Istanbul University Licensed Software). Data normality was assessed using the Kolmogorov–Smirnov test. The Chi-square test was used for categorical variables. One-way analysis of variance was performed for the Schirmer test, TBUT, and goblet cell density. A value of p < 0.05 was significant.

## RESULTS

Nineteen (42.2%) individuals were male and 26 (57.8%) individuals were female in the contact lens wearing group. Nine (42.9%) male participants and 12 (57.1%) female participants constituted the control group. The mean usage duration of contact lenses in all three groups was not significantly different, although contact lens wearing history was longer in the RGP group (p = 0.089) (Table 1).

**Figure 1:**
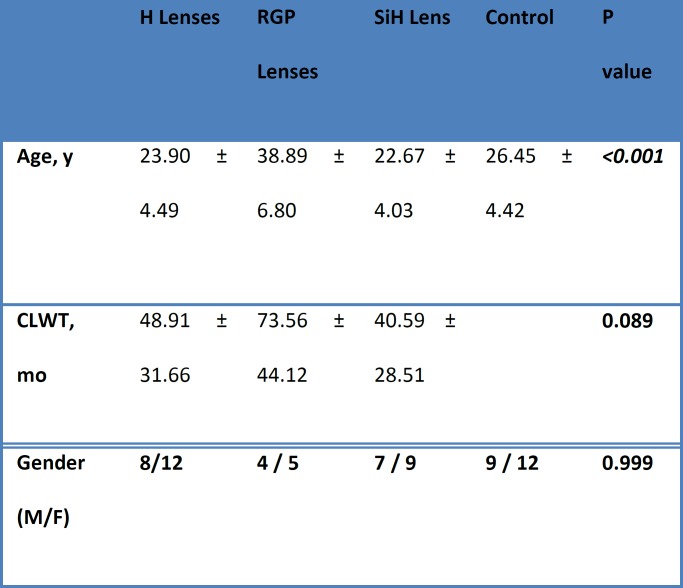
Impression Cytology Grade 0

**Figure 2:**
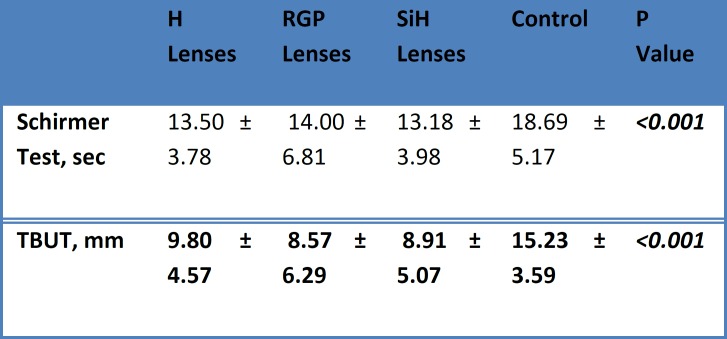
Impression Cytology Grade 2

The mean values of the Schirmer test were statistically significantly different between the groups (p < 0.001). The mean values of the TBUT were statistically significantly longer in the control group than in the H, SiH, and RGP groups (p < 0.001) (Table 2). In the CIC assessment, grade 0 was most frequent in the control group (66.7%) and least frequent in the SiH lens group (40.6%; Fig. 1); grade I was least frequent in the control and RGP groups (33.3%) and most frequent in the SiH lens group (40.6%); and grade 2 was most frequent in the SiH lens group (18.8%; Fig. 2). A statistically significant difference was not detected between groups (Table 3).

**Table 1:** Patients’ Demographics

	**H Lenses**	**RGP Lenses**	**SiH Lens**	**Control**	**P value**
**Age, y**	23.90 ± 4.49	38.89 ± 6.80	22.67 ± 4.03	26.45 ± 4.42	***<0.001***
**CLWT, mo**	48.91 ± 31.66	73.56 ± 44.12	40.59 ± 28.51		**0.089**
**Gender (M/F)**	**8/12**	**4 / 5 **	**7 / 9 **	**9 / 12**	**0.999**

CTLWT, contact lens wearing time; F, female; H, hydrogel; M, male; RGP, rigid gas-permeable permeable; SiH, silicone hydrogel.

**Table 2:** Schirmer Test and Tear Break-up Time

	**H Lenses**	**RGP Lenses**	**SiH Lenses**	**Control**	**P Value**
**Schirmer Test, sec**	13.50 ± 3.78	14.00 ± 6.81	13.18 ± 3.98	18.69 ± 5.17	***<0.001***
**TBUT, mm **	**9.80 ± 4.57 **	** 8.57 ± 6.29 **	** 8.91 ± 5.07 **	**15.23 ± 3.59 **	***<0.001***

H, hydrogel; RGP, rigid gas-permeable; SiH, silicone hydrogel; TBUT, tear break-up time.

## DISCUSSION

Conjunctival cytologic studies of patients wearing contact lenses demonstrate the development of squamous metaplasia on the ocular surface (3,5,6,8,9). This change is the primary cause of contact lens intolerance and occurs in most ocular surface disorders, particularly in dry eye (10). As morphological changes of squamous metaplasia progresses, the conjunctival epithelium loses its function. Moreover, the epithelial cell size increases, the nucleus-to-cytoplasm ratio decreases, and the number of goblet cells decreases (1,6,10).

Epithelial cells are small with a nucleus cytoplasm ratio of 1/2 and the goblet cell density is high (13 goblet cells).

Larger polygonal epithelial cells are evident with a further decrease in nucleus-to-cytoplasm ratio of 1/4 to 1/5; only two goblet cells are visible.

The prevalence and severity of cytological alteration were significantly higher in symptomatic contact lens wearers than in asymptomatic contact lens wearers. It increased significantly in parallel with the duration of lens wear (3).

Conjunctival epithelial changes in contact lens wearers may be caused by anoxia, inflammation, an immunological or infectious origin, and chronic mechanical irritation; however, these mechanisms are not clearly understood. Knop and Brewitt (6) defined these mechanisms by metabolic inflammation and chronic mechanical irritation. According to the chronic irritation theory, contact lenses cause epithelial stress with blinking, and result in squamous metaplasia and snakelike chromatin changes. This theory can explain the changes in conjunctival epithelia, secondary to contact lens wearing. In the asymptomatic group of soft contact lens wearers, snakelike chromatin was present in 13 of 14 patients, based on limbal bulbar conjunctival samples, which were taken from the 12-o’clock position by CIC. In addition, fewer amounts at the 6-o’clock position and rarely in the nasal and limbal conjunctiva with a same appearance have been seen (6).

**Table 3:** Distribution of the Groups, Based on the Nelson Grading System

**Grading **	**H Lenses** **(n=40)**	**RGP Lenses (n=18)**	**SiH Lenses** **(n=32)**	**Control** **(n=2)**
**Grade 0**	21 (52.5%)	9 (50.0%)	13 (40.6%)	**14 (66.7%)**
**Grade 1**	14 (35 %)	6 (33.3%)	13 (40.6%)	**7 (33.3%)**
**Grade 2**	5 (12.5%)	3 (16.7%)	6 (18.8%)	**0 ( 0%)**
**P.value**	**0.445 **			

H; hydrogel, n= number of eyes, RGP; rigid gas permeable, SiH; silicone hydrogel,

Knop and Brewitt (6) report that the mechanical effect of contact lenses on the conjunctiva can cause conjunctival epithelial changes. We agree with Knop and Brewitt because contact lens and superior eyelid movements cause mechanical irritation and increase metaplastic changes, primarily in the upper bulbar conjunctiva.

Adar et al. (5) determined that, based on the Nelson grading system, 90% of typical patients are in grade 0 and 10% of patients are in grade 1. Aragona et al. (4) and Anshu et al. (11) evaluated cytologic changes in patients wearing soft or gas-permeable contact lenses and found that the cytologic changes were more advanced in patients wearing soft contact lenses. In these two studies, the impression cytologic samples were compared with those of control groups, which was the same procedure used in the present study. In our study grade 0 was most frequent in the control group (66.7%) and least frequent in the silicone hydrogel lens group (40.6%); grade I was least frequent in the control and RGP groups (33.3%) and most frequent in SiH lens group (40.6%); and grade 2 was most frequent in SiH lens group (18.8%). No statistically significant difference was detected between groups.

**Table 4:** Mean Goblet Cell Density in the Control Group and Contact Lens Groups

	**H Lenses**	**RGP Lenses**	**SiH Lenses**	**Control**
**Cell density, cell/mm² **	537 ± 243	455 ± 205	463 ± 231	**529 ± 245**
	**p= 0.462**			

Paschides et al. (12) reported that the density of goblet cells was 510 ± 563 cells/mm2 in the superior bulbar conjunctiva of healthy individuals. Çakmak et al. (8) found a goblet cell density of 433.25 ± 184 cells/mm2, which was obtained from the superior limbal conjunctiva. Yeo et al. (13) reported that goblet cell density alone is not a useful diagnostic indicator of tear film instability in healthy eyes, but it is a good indicator in the diagnosis of ocular surface diseases. We found a goblet cell density of 529 ± 245 cells/mm2 in samples that we obtained from the superior limbal conjunctiva. This result was similar to that of studies by Paschides et al. (12), and Çakmak et al. (8). Lievens et al. (9) evaluated the response of the ocular surface to extended contact lens wear by comparing SiH lens with H lens at baseline and for 6 months after initiation of lens wear; they found increased goblet cell density in individuals wearing disposable H lenses and SiH lenses. In the current study, asymptomatic contact lens wearers using H, SiH, or RGP lens were compared to healthy individuals (i.e., non-wearers). There was no statistically significant difference in goblet cell density between the healthy contact lenses wearers and the three different types of contact lens wearers.

Aragona et al. (4) found that the average TBUT was 12.84 seconds in the control group but 8.36 seconds in soft contact lens wearers, and the Schirmer test result was 16.76 mm in the control group and 9.80 mm in soft contact lens wearers. Çakmak et al. (8) found the average TBUT and Schirmer test results were 12.26 seconds and 15.59 mm, respectively, in the control group, and 8.66 seconds and 9.60 mm, respectively, in the group wearing soft contact lens for 13–48 months.

The current study showed a statistically significant difference between contact lens wearers and the control group for the Schirmer test and TBUT results. Our findings were compatible with the findings of previous studies (4,8). The mechanism affecting the tear film is not explicit, but contact lens can cause mechanical irritation (i.e., microtrauma) in the conjunctiva and subclinical inflammation in the conjunctiva and conjunctival glands.

In conclusion, tear film stability is affected in contact lens wearers, compared to healthy non-wearers. In addition, contact lens may cause some cytologic changes on the ocular surface. These may occur in asymptomatic eyes and in symptomatic eyes. Conjunctival impression cytology is a simple and noninvasive technique that may be used to evaluate the ocular surface with regard to intolerance to contact lenses.
